# The link between Mitochondria and Sarcopenia

**DOI:** 10.1007/s13105-024-01062-7

**Published:** 2025-02-19

**Authors:** Nurul Tihani Kamarulzaman, Suzana Makpol

**Affiliations:** Department of Biochemistry, Faculty of Medicine, Universiti Kebangsaan Malaysia, Jalan Yaacob Latif, Bandar Tun Razak, Cheras, Kuala Lumpur, 56000 Malaysia

**Keywords:** Sarcopenia, Mitochondria, Muscle ageing, Oxidative stress, Mitochondrial protein interactions

## Abstract

Sarcopenia, a widespread condition, is characterized by a variety of factors influencing its development. The causes of sarcopenia differ depending on the age of the individual. It is defined as the combination of decreased muscle mass and impaired muscle function, primarily observed in association with ageing. As people age from 20 to 80 years old, there is an approximate 30% reduction in muscle mass and a 20% decline in cross-sectional area. This decline is attributed to a decrease in the size and number of muscle fibres. The regression of muscle mass and strength increases the risk of fractures, frailty, reduced quality of life, and loss of independence. Muscle cells, fibres, and tissues shrink, resulting in diminished muscle power, volume, and strength in major muscle groups. One prominent theory of cellular ageing posits a strong positive relationship between age and oxidative damage. Heightened oxidative stress leads to early-onset sarcopenia, characterized by neuromuscular innervation breakdown, muscle atrophy, and dysfunctional mitochondrial muscles. Ageing muscles generate more reactive oxygen species (ROS), and experience decreased oxygen consumption and ATP synthesis compared to younger muscles. Additionally, changes in mitochondrial protein interactions, cristae structure, and networks may contribute to ADP insensitivity, which ultimately leads to sarcopenia. Within this framework, this review provides a comprehensive summary of our current understanding of the role of mitochondria in sarcopenia and other muscle degenerative diseases, highlighting the crucial need for further research in these areas.

## Introduction

The increasing number of older individuals in society signifies progress in socioeconomic terms and reflects demographic changes in developed countries. Ageism has been shown to significantly harm health, with a vast majority of studies (95.5%) and ageism-health associations (74%) revealing worse outcomes (Chang et al. 2020). These detrimental effects were observed in all forty-five countries examined, spanning 11 different health domains over a 25-year period, with the frequency of findings increasing over time. Interestingly, less-developed countries exhibited a higher rate of significant ageism-health associations compared to more-developed nations, likely due to fewer healthcare resources for older populations. By 2030, Malaysia is projected to transition into an ageing nation, with 15% of its total population comprising elderly individuals (Md Nor and Ghazali 2021). This demographic shift can be attributed to declining fertility and mortality rates, along with improvements in education, healthcare, and employment opportunities, which have contributed to increased life expectancy and the growth of the ageing population in Malaysia (Abdul Rashid et al. 2016; Md Nor and Ghazali 2021; Tey et al. 2016). Older adults with lower education levels were especially prone to health issues related to ageism. A systematic review by Chang et al. (2020) also highlighted that ageism crosses demographic boundaries, impacting individuals regardless of age, gender, or race. Decisions influenced by ageism, often unconsciously, restricted access to care, and older patients were frequently excluded from relevant clinical trials. The economic impact of ageism is also considerable, with an estimated $63 billion in ageism-related healthcare costs in the U.S. annually (Chang et al. 2020). On a global scale, ageism is linked to approximately 6.33 million cases of depression in older adults (Chang et al. 2020). Reducing ageism could not only enhance health outcomes but also prove to be cost-effective, particularly in less-developed countries facing an increase in elderly populations (Chang et al. 2020).

While ageing may seem detrimental to individuals, it is an adaptive response to the external environment and should be distinguished from external damage to the body, which can have detrimental effects on an individual’s physiological and overall health (Ahn et al. 2022; Barja 2013; Dillon et al. 2012). Throughout an individual’s lifetime, ageing is characterized by a gradual decrease in maximum physiological capacities (Barja 2013; Dillon et al. 2012). At the molecular level, ageing is also thought to result from the accumulation of random damage to cellular components such as DNA, proteins, and lipids. Ageing is also believed to result from a lifetime accumulation of stochastic damage to tissues and cellular components such as DNA, proteins, and lipids at the molecular level; suppose the damage is at the root of ageing. In that case, the main challenges are determining which cellular components are most likely involved in the primary mechanisms underlying functional deterioration and dissecting and understanding the highly networked pathways involving damage and repair (Akbari et al. 2019).

Normal ageing is seen as a decrease in bone and muscle mass and an increase in adiposity. Regression in muscle mass and muscle strength leads to the risk of fractures, frailty, reduced quality of life, and loss of independence (Amarya et al. 2018; Carter et al. 2015; Del Campo et al. 2018; Drescher et al. 2015; Fazelzadeh et al. 2016; Pharaoh et al. 2021; Rahman and Quadrilatero 2021; Uutela et al. 2018; Wada et al. 2019). These alterations in the musculoskeletal system are a result of both the natural ageing process and, to some extent, reduced physical activity. While physical inactivity can significantly contribute to muscle atrophy, the declines associated with ageing itself are largely driven by internal physiological changes, including hormonal shifts, inflammation, and alterations in muscle fibres, such as an increase in slow-twitch fibres. This distinction is important because it highlights that even active older adults can experience muscle loss and weakening due to the ageing process alone. However, when physical inactivity is present, it can further worsen these effects, accelerating muscle loss and leading to more pronounced declines in function. Thus, although ageing and muscle disuse are distinct factors, they often interact, compounding the challenges of maintaining muscle mass and strength in older individuals. As a result, age-related loss of muscle mass and function, particularly in terms of muscle strength and gait speed, is closely linked to sarcopenia (Amarya et al. 2018; Drescher et al. 2015; Fazelzadeh et al. 2016; Wada et al. 2019). Sarcopenia, which is defined as the loss of muscle mass and function, is not an inevitable consequence of ageing but can develop in some individuals due to a combination of genetic, environmental, and lifestyle factors (Cruz-Jentoft and Sayer 2019). Sarcopenia can be classified into two categories: primary sarcopenia, which is solely due to ageing, and secondary sarcopenia, which is influenced by factors such as physical inactivity, malnutrition, or chronic disease. The underlying mechanism of muscle atrophy is an imbalance between protein synthesis and protein degradation (Drescher et al. 2015; Ormsbee et al. 2014; Patel et al. 2013; Ramy et al. 2020). Additionally, sarcopenia is commonly associated with chronic diseases such as cancer, chronic heart failure (HF), chronic obstructive pulmonary disease, chronic kidney disease (CKD), cystic fibrosis, liver cirrhosis, Crohn’s disease, rheumatoid arthritis (RA), stroke, and many neurodegenerative diseases as well as in human immunodeficiency virus or acquired immune deficiency syndrome, malaria, and tuberculosis (Drescher et al. 2015; Farkas et al. 2013; Von Haehling and Anker 2014).

### Degeneration of muscles

Skeletal muscle force-generating ability declines with age, based on genetic, nutritional, environmental factors, and lifestyle preferences, affecting physical mobility and everyday activities (Amarya et al. 2018; Carter et al. 2015; Del Campo et al. 2018; Drescher et al. 2015; Pharaoh et al. 2021; Rahman and Quadrilatero 2021; Wada et al. 2019). Sarcopenia, cachexia, and malnutrition are the leading causes of muscle wasting and affect millions of older people and patients (Drescher et al. 2015; Farkas et al. 2013; Von Haehling and Anker 2014). Reduced molecular and architectural muscle frame changes, such as a reduction in the number of muscle fibres, lead to sarcopenia, which reduces muscle quality, functions, strength, and stamina. Skeletal muscle includes 40% of body mass in young, healthy people and is essential for locomotion and whole-body metabolism. At 45 years of age, the rate of muscle mass loss begins to slow, resulting in a loss of 0.5–1.4% of muscle mass each year. Muscle functions begin to decline significantly as people age, particularly around 60 years of age. Sarcopenia can cause physical deterioration, as well as the emergence of metabolic disorders such as Type 2 diabetes and obesity, plus a higher chance of falling (Amarya et al. 2018; Carter et al. 2015; Del Campo et al. 2018; Fazelzadeh et al. 2016; Pharaoh et al. 2021).

The overall number of muscle fibres is reduced due to a decline in the active ability of cells to generate protein. Muscle cells, fibres, and tissues shrink, resulting in a lack of muscle power, bulk, and strength in all large muscle groups, such as the deltoids, biceps, triceps, hamstrings, gastrocnemius (calf muscle), and others. The supportive cartilage of joints also wears out. Usually, cartilage serves as a shock absorber and a gliding agent, preventing bone friction injuries, but with sarcopenia, connective tissue elements stiffen and fibrosis, reducing the range of motion and making motions less effective (Amarya et al. 2018; Carter et al. 2015; Jackson et al. 2022). Patients with muscle atrophy show decreased muscle strength and reduced quality of life caused by lower activity and increased exercise intolerance. In sarcopenic patients, muscle wasting is frequently associated with bone loss, leading to a higher risk of hip and other fractures. Hip fracture also results in loss of musculature because of disuse muscle atrophy. All these situations lead to increased morbidity and mortality in patients; thus, developments in biomarkers and treatments that can enhance patients’ lives are required (Drescher et al. 2015; Ormsbee et al. 2014; Patel et al. 2013). Physical fitness declines and toxins and contaminants accumulate in the body and tissues as people age and due to lifestyle changes (Amarya et al. 2018; Del Campo et al. 2018). Age-related alterations in the brain exacerbate physiological changes in the muscles to a certain extent. As a result of deterioration in nerve function and nerve conduction, most muscle movements become less effective and receptive with age. Some of the cellular modifications observed in aged muscle cells are accumulated intra or extracellular lipids, misfolding of structural and contractile proteins, and mitochondrial dysfunction. Chemicals, toxins, and waste products generated by the body have a more significant impact on DNA. This entire mechanism makes cells more vulnerable (Amarya et al. 2018; Del Campo et al. 2018).

Muscle fibres denervation is a significant factor affecting the ageing process of muscles. Throughout an individual’s adult life, transient events of denervation and reinnervation drive changes, such as shifts in fibre type and the grouping of muscle fibres within motor units. These changes lead to a decline in motor control and reduced increases in muscle blood flow during contractions as individuals age (Anagnostou and Hepple 2020). In advanced age, denervation has a more pronounced negative impact, contributing to mobility limitations. Persistent denervation accelerates muscle atrophy and contractile dysfunction. Therefore, understanding the mechanisms that cause muscle fibre denervation with age is crucial in developing strategies to address the impairment of ageing muscles. The alterations occurring in muscle fibres, the innervating motor neuron, and the neuromuscular junction—the specialized synapse connecting these structures—may play a role in the denervation of skeletal muscle during the ageing process (Anagnostou and Hepple 2020; Deschenes 2004).

Muscle deficiency also can be caused by an imbalance in catabolism and anabolism, such as protein degradation and synthesis. An imbalance causes muscle atrophy in protein synthesis and degradation (Drescher et al. 2015; Jackson et al. 2022; Ormsbee et al. 2014; Patel et al. 2013). The activation of the ubiquitin-proteasome–system (UPS) is one of the major protein degradation pathways that play a role in developing muscle wasting (UPS). The researchers discovered that there was a gradual progression of muscle wasting with an early onset, which was linked to elevated serum levels of cytokines such as interleukin-6 (IL-6) (Brooks and Myburgh 2014; Drescher et al. 2015; Farkas et al. 2013; Jackson et al. 2022; Ormsbee et al. 2014; Patel et al. 2013; Rosenberg 2011; Von Haehling and Anker 2014). It also has been revealed that high levels of TNF-α cause muscle weakness and that plasma amounts of the enzyme visfatin are considerably higher in patients who have had an ischemic stroke (Drescher et al. 2015) or cardiovascular-metabolic disorders. The involvement of declining mitochondrial function in age-related transformations in skeletal muscle has been broadly studied to understand the grounds of sarcopenia. Furthermore, there is active research and debate on how skeletal muscle ageing affects mitochondria and how ageing mitochondria affects skeletal muscle function. This review will discuss recent discoveries in the area of muscle mitochondria and ageing and how age-related changes in oxidative capacity, protein turnover, and mitochondrial dysfunction contribute to ageing and sarcopenia.

### Role of mitochondria in muscle homeostasis

Mitochondria are involved in several critical cellular processes, including energy synthesis, calcium signaling, amino acid and nucleotide metabolism, fatty-acid catabolism, ion homeostasis, reactive oxygen species (ROS) generation, and apoptosis (Ahn et al. 2022; Anagnostou and Hepple 2020; Calvani et al. 2013; Gioran and Chondrogianni 2020; Joseph et al. 2016; Rahman and Quadrilatero 2021). Any of these mechanisms can be disrupted, affected by the quantity and consistency of mitochondria, and potentially triggering a chain of adverse events within the cell. Mitochondria are primarily clustered in distinct regions of the muscle. There are three distinct fibre groups that make up skeletal muscles, as discovered by myosin ATPase histochemistry and electrophoretic studies of myosin heavy chain isoforms. Type I fibres are the smallest, most mitochondria abundant but give the least amount of force, making them fatigue resistant. Type II fibres are more prominent and have fewer mitochondria than Type I fibres, but they can produce more force and are more fatigable. Multiple myosin heavy chain isoforms have been reported to be co-expressed in muscle fibres, resulting in mixed or hybrid fibre groups. Hybrid fibres are more common in aged tissue, adding to the difficulty of fibre-type classification in older people’s muscles (Amarya et al. 2018; Carter et al. 2015; Del Campo et al. 2018; Rahman and Quadrilatero 2021). Fast-twitch muscle fibres provide much more evidence of age-related musculoskeletal improvements than slow-twitch muscle fibres. When people get older, their overall tissue’s water content reduces, and the lack of hydration contributes to inelasticity and stiffness. Muscle modifications will occur because of changes in the basal metabolic rate and slowing metabolism due to physiological ageing. As a result, proteins are replaced by fatty tissue, making muscle less efficient in function (Del Campo et al. 2018; Rahman and Quadrilatero 2021).

Mitochondria form complex interconnected networks within skeletal muscle that are critical for efficient energy production and distribution, and they contribute to the formation of ROS along with other reactions. These networks are localized in regions of the cell that have high energy demands, particularly nearat sites of ATP consumption, such as the myofibrils and the sarcoplasmic reticulum. These regions are referred to as “sensitive” because they are critical for muscle contraction and calcium handling, thus necessitating efficient energy production (Gioran and Chondrogianni 2020; Pharaoh et al. 2021; Rahman and Quadrilatero 2021). Mitochondrial respiration and ATP production are primarily driven by cytosolically derived ADP, which is produced during ATP-consuming reactions. In a resting myocyte, ATP demand is low, therefore, substrate oxidation and electron transport are minimized and controlled by a high proton motive force across the inner mitochondrial membrane. In this condition, electrons can convert oxygen into ROS (Rahman and Quadrilatero 2021). However, it is important to note that ROS production is not limited to low ATP demand conditions. During periods of high ATP demand, such as exercise, the increased activity of the electron transport chain can elevate the likelihood of ROS generation due to heightened substrate oxidation and electron transfer. The extent of ROS production is influenced by factors such as electron leakage and the functional efficiency of the electron transport chain complexes, which can either favor or mitigate ROS formation (Murphy et al. 2022). Thus, both resting and active states can contribute to ROS production, depending on mitochondrial function and ATP requirements. When myocytes contract, myosin ATPase generates free ADP, which enters mitochondria to interact with ATP synthase. This increase in ADP concentration stimulates ATP synthesis by ATP synthase, which consumes protons from the intermembrane space. This action dissipates the proton gradient, thereby enhancing the driving force for electron transfer through the electron transport chain, including cytochrome oxidase (Rahman and Quadrilatero 2021). The presence of ADP further promotes the activity of the electron transport chain by facilitating the dephosphorylation of cytochrome c, which is critical for efficient electron transfer.

Consequently, as muscle cells contract, oxygen consumption and ATP synthesis increase, while mitochondrial ROS production per unit of oxygen (O_2_) consumption diminishes (Carter et al. 2015; Jackson et al. 2022; Joseph et al. 2016; Pharaoh et al. 2021; Rahman and Quadrilatero 2021). Alterations in mitochondrial protein interactions, cristae structure, and networks may also play a role in ADP insensitivity. The content of cardiolipin, an inner mitochondrial membrane phospholipid, decreases in skeletal muscle mitochondria with age, which decreases the folding of mitochondrial cristae and decreases ETS efficiency (Gioran and Chondrogianni 2020; Pharaoh et al. 2021; Rahman and Quadrilatero 2021). Myosin ATPase produces free ADP as muscle cells contract, which interacts with ATP synthase, further influencing mitochondrial function and dissipating the proton gradient and allowing an increased rate of electron transfer to cytochrome oxidase. Therefore, oxygen consumption and ATP synthesis rise while mitochondrial ROS input per unit of O_2_ consumption falls (Table 1) whether per organelle or unit of muscle mass (Carter et al. 2015; Jackson et al. 2022; Joseph et al. 2016; Pharaoh et al. 2021; Rahman and Quadrilatero 2021). While ROS can have signaling functions and potential harmful effects, it is important to clarify that these effects are not directly linked to the process of muscle contraction. Instead, ROS can contribute to the opening of the mitochondrial permeability transition pore (mtPTP), which causes organelle swelling and allows pro-apoptotic proteins to be released into the cytosol under certain conditions, such as oxidative stress or cellular damage. When these factors are released, they cause apoptotic signaling and, eventually leading to myonuclear degradation and muscle atrophy (Jackson et al. 2022; Rahman and Quadrilatero 2021). Furthermore, mitochondria are also involved in calcium sequestration, which can trigger intracellular signaling by modulating cytosolic calcium levels. Thioredoxin-dependent peroxide reductase (PRDX3) overexpression in mitochondria was also proven by Ahn et al. (2022) to prevent mitochondrial calcium buffering capacity in Sod1KO mice (mice lacking Cu/Zn-superoxide dismutase) from being significantly impaired. In short, mitochondria plays a variety of functions in myocytes, and the diversity of these functions is considered when studying mitochondrial dysfunction and its relationship to muscle mass (Ahn et al. 2022; Jackson et al. 2022; Pharaoh et al. 2021; Rahman and Quadrilatero 2021).

**Table 1:** The role of mitochondria in sarcopenia (in-vivo study in human and animal)

Authors & References	Involvement of Mitochondria	Findings
Ahn et al. (2022)	Oxidative stress	• The study measured mitochondrial calcium retention capacity (CRC) of isolated skeletal muscle mitochondria by challenging the mitochondria with sequential additions of calcium chloride until the opening of permeability transition pore.
		• Mitochondria buffers cytosolic calcium ions, regulating the calcium concentration in cytoplasm.
		• The results demonstrate that scavenging mitochondrial hydrogen peroxide prevents contractile dysfunction and attenuates muscle atrophy independent of protection against loss of neuromuscular junction (NMJ) structure and function in a redox-dependent sarcopenia.
Fazelzadeh et al. (2016)	Glucose and fatty acid metabolism	• A study using mice showed that ageing affects muscle glucose and fatty acid metabolism, whereas a study in rats indicated that ageing leads to muscle group-specific perturbations in lipid and glucose metabolism consistent with mitochondrial dysfunction.
		• In humans, it was found that changes in lipid content and oxidative activity in skeletal muscle during ageing are related to a shift in muscle fibre type.
		• Decreased expression of genes resulted in mitochondrial functions resulted in impaired mitochondrial function and lower number of mitochondria in muscle of older subjects, which effected lower levels of ATP, ADP, branched chain amino acids and acylcarnitine.
Choi et al. (2016)	Decline in bioenergetics	• The study assessed the rate of maximum in vivo oxidative capacity of skeletal muscle by 31-Phosphorus Magnetic Resonance Spectroscopy (31P-MRS) inwhich is not affected by postural stability or balance, and therefore represents a more direct measure of muscle mitochondrial function.
		• Kinetic phosphocreatine (kPCr) is the rate of utilization and replenishment of phosphocreatine, a high-energy phosphate compound in muscles during exercise. It is an indicator of muscle energy metabolism and mitochondrial function.All gait speeds, kinetic phosphocreatine (kPCr), and peak oxygen uptake (VO2) was lower with older age. Independent of age, sex, height, and weight, both kPCr and peak VO2 were positively and significantly associated with fast pace and long-distance walking but only peak VO2 and not kPCr was significantly associated with usual gait speed over 6 min.
Kumar et al. (2017)	Decline in bioenergetics/OXPHOS capacity	• Metabolic syndrome has been shown to disrupt mitochondrial function, depress skeletal muscle OXPHOS, and lead to ectopic lipid storage in skeletal muscle.
		• Aberrant mitochondrial function, either acquired or genetic, could contribute to the development or progression of cardiac dysfunction.
		• Diabetes and metabolic syndrome result in mitochondrial abnormalities in myocardium, such as impaired biogenesis and metabolism of fatty acid, which lead to reduced substrate flexibility, energy efficiency, and eventually, diastolic dysfunction.
Lee et al. (2018)	Calcium homeostasis	• The results strongly support the critical role of mito-Ca^2+^ homeostasis regulated by Ca^2+^ transfer through the endoplasmic reticulum–mitochondria contact sites (ERMCS) in mediating PINK1 function in neuromuscular tissues in an intact animal.
		• Interestingly, matrix Ca^2+^ has also been suggested as an intrinsic signal controlling mitochondrial transport in neuronal axons. These data raise the intriguing possibility that matrix Ca^2+^ may coordinate mitochondrial motility and shape.
		• The results further support the Miro signaling pathway as an important regulator of mito-Ca^2+^ homeostasis mediated by ERMCS in neurons.
Pharaoh et al. (2021)	ADP Sensitivity	• The study found no age-related change in ADP sensitivity kinetics for OCR, leak respiration, OXPHOS capacity, CII‐linked respiration, CIV‐linked respiration, or OXPHOS coupling efficiency.
		• Denervation does not induce adenosine diphosphate insensitivity but results in progressive muscle atrophy and increased basal hydroperoxide production from isolated mitochondria.
		• The study found that there is no difference in ADP sensitivity kinetics for ROS production, but loss of innervation does surprisingly decrease the ability of permeabilized fibres to produce hydroperoxides after addition of antimycin A and during maximal stimulation conditions.
		• Together, these data show that loss of innervation does not induce mitochondrial ADP insensitivity.
Tsakiri et al. (2019)	Interventions of ubiquitin-proteasome system (UPS)	• In *Drosophila melanogaster,* knock-down of various proteasomal subunits, caused significant fragmentation of the mitochondrial network that was accompanied by reduction in mitochondrial respiration.
Ugun-Klusek et al. (2017)	Interventions of ubiquitin-proteasome system (UPS)	• Cortical neurons from mice lacking a proteasome subunit manifested a fragmented mitochondrial network and respiratory complex I defects.
		• The proteasome inhibitor lactacystin increased mitochondrial ROS production and oxidative stress and diminished the mitochondrial membrane potential in mouse cortical neurons.
Wang et al. (2022)	Proteostatic stress	• The study found that several upregulated genes in the ‘mitochondrial inner membrane’ gene ontology group are involved in mitochondrial protein import, including *TOMM40*, *TOMM5*, *TOMM34*, *TIMM8A1*, *TIMM10*, *TIMM44*, *HSPA9*, *DNAJA3*, and *CHCHD10*
		• This supports the existence of a retrograde regulatory mechanism in the skeletal muscle that promotes mitochondrial protein import.
		• The reminiscent of mitochondrial Unfolded Protein Response (mtUPR) and mitoCPR mechanisms are activated to cope with increased proteostatic stress inside the mitochondria of *C. elegans* and *S. cerevisiae.*
Wrobel et al. (2015)	Interventions of ubiquitin-proteasome system (UPS)	• The study challenged the mitochondrial protein import machinery by mutations in genes encoding for its components, specifically the inner membrane translocase, TIM23. All insults induced proteasome activity and reduced translation in the cytosol. This response was named “unfolded protein response activated by mistargeted proteins” or UPRam and it was suggested to maintain cellular homeostasis.
		• Notably, UPRam induction also rendered the cells more resistant to heat-shock, thus possibly indicating the activation of an additional proteostatic response.
Sliter et al. (2018)	Parkin and PINK1 mitigate STING-induced inflammation	• This study elucidates the critical role of Parkin in maintaining cellular homeostasis and preventing inflammation. Understanding the mechanisms by which Parkin and PINK1 interact with the STING pathway opens new avenues for therapeutic interventions in Parkinson’s disease and potentially other neurodegenerative disorders characterized by mitochondrial dysfunction and inflammation.
		• This study reports a strong inflammatory phenotype in both *Prkn −/−* and *Pink1 −/−* mice following exhaustive exercise and in *Prkn −/−*;*mutator* mice, which accumulate mutations in mitochondrial DNA (mtDNA).
		• To acutely stress mitochondria, 12-week-old mice were exercised until exhausted for three consecutive days. No differences in the time to exhaustion were observed among wild-type, *Prkn −/−* or *Pink1 −/−* mice as circulating mtDNA levels and ratios of mtDNA to nuclear DNA were higher in 40-week-old *Prkn −/−*;*mutator* mice compared to wild-type, *mutator* or *Prkn −/−* mice, and loss of STING did not rescue the increase.
		• Inflammation resulting from either exhaustive exercise or mtDNA mutation is completely rescued by concurrent loss of STING, a central regulator of the type I interferon response to cytosolic DNA. The loss of dopaminergic neurons from the substantia nigra pars compacta and the motor defect observed in aged *Prkn −/−*;*mutator* mice are also rescued by loss of STING.
		• Humans with mono- and biallelic *PRKN* mutations also display elevated cytokines. These results support a role for PINK1- and parkin-mediated mitophagy in restraining innate immunity.
Song and Cortopassi (2015)	Interventions of ubiquitin-proteasome system (UPS)	• Accordingly, mice carrying a deletion of Ndufs4, a mitochondrial complex I subunit, exhibited reduced proteasome activities in their midbrain

Mitochondrial morphology in mature myofibres is divided into two distinct but interconnected species, each with its size and morphological parameters. In adult muscle fibres, sub-sarcolemma (SSM) and intermyofibrillar (IMF) mitochondria are thought to form an integrated network that adapts cell-type-specific morphology and fuse and fission (Akbari et al. 2019; Carter et al. 2015; Del Campo et al. 2018; Giedt et al. 2016; Gioran and Chondrogianni 2020; Jackson et al. 2022; Pharaoh et al. 2021; Rahman and Quadrilatero 2021). Mitofusin 1 (MFN1), mitofusin 2 (MFN2), and optic atrophy type 1 are essential factors in the fusion phase (OPA1) (Rahman and Quadrilatero 2021; Rodger et al. 2018). Fusion, fission, and mitophagy are intertwined mechanisms that define mitochondrial morphology and dynamics (Akbari et al. 2019; Gioran and Chondrogianni 2020; Rahman and Quadrilatero 2021; Sebastian et al. 2017). Fusion events become less frequent in muscle fibres than in undifferentiated myoblasts, resulting in a more specialized and durable matrix domain complementation. These changes can be attributed to the highly compartmentalized architecture of the muscle fibre, which offers few possibilities for the movement of the mitochondria to join and separate from other individual mitochondria (Del Campo et al. 2018). Flexor digitorum brevis (FDB) muscles of 1-month-old mice have more elongated and less organized mitochondria than the muscle fibres of adult mice. In the adults, the majority of the globular intermyofibrillar (IMF) mitochondria are located in the I band, in opposition to the terminal cisternae of the sarcoplasmic reticulum and the T-tubule where the calcium release units or triads are formed (Del Campo et al. 2018; Rossi et al. 2022).

The cell’s ability to transcribe, translate, and import new proteins into pre-existing organelles is crucial for mitochondrial synthesis (Akbari et al. 2019; Carter et al. 2015; Jackson et al. 2022; Rahman and Quadrilatero 2021). The nuclear genome contains most genes that code for thousands of mitochondrial proteins. However, mtDNA is home to thirteen crucial genes that code for ETC components (Carter et al. 2015; Gioran and Chondrogianni 2020). Electron transport chain (ETC) is composed by several protein complexes localized in the inner mitochondrial membrane and primarily responsible for ATP production via aerobic respiration. The chain is composed of four primary protein complexes (I–IV) and two mobile electron carriers, coenzyme Q (ubiquinone) and cytochrome c, that mediate inter-complex electron transport. The process begins when NADH is oxidized by complex I (NADH dehydrogenase or ubiquinone) to donate two electrons to form FMNH_2_, which are then passed through iron-sulphur (Fe-S) clusters. These electrons are then transmitted to ubiquinone and reduced to ubiquinol. In the process, Complex I pumps four protons (H^+^) from the mitochondrial matrix into the intermembrane space generating an electrochemical proton gradient (Ahmad et al. 2024). Complex II (succinate dehydrogenase), a second entry point for electrons, oxidizes succinate to fumarate and feeds the resulting electrons via FADH_2_ into iron-sulphur clusters of Complex III in an analogous series of steps as Complex I; however, it does not translocate protons across the mitochondrial inner membrane and hence does not help in pumping protons out of the matrix directly between internal quinone layers (Q-cycle). Ubiquinone/ubiquinol (Coenzyme Q), in its reduced form ubiquinol, shuttles electrons from both Complex I and Complex II to Complex III (cytochrome bc1 complex or cytochrome c reductase). Cytochrome b reduces and oxidizes ubiquinol and takes electrons from an intermediate in this process to pass them along into Complex IV (cytochrome c oxidase), where a two-electron transmission stacking site involving cytochromes b, c1, c, iron-sulphur proteins. It pumps four protons from the matrix into intermembrane space, thus contributing towards proton gradient (Ahmad et al. 2024). Cytochrome c is a small, mobile protein that can only transport one electron at a time and passes it to Complex IV, cytochrome c oxidase. Complex IV finishes off the electron transport chain by pumping electrons to molecular oxygen (O_2_), the ultimate electron acceptor of aerobic respiration. Oxygen is reduced to water in this step, with four more protons pumped across the membrane. The generated proton gradient is utilized by the ATP synthase, or Complex V, to generate ATP through a process referred to as chemiosmosis. The protons re-enter the mitochondrial matrix through the ATP synthase, transferring their energy to the production of ATP from ADP and inorganic phosphate, Pi. Four protons passing through the ATP synthase result in the generation of one ATP. Thus, the ETC is absolutely essential in the process of cellular energy production, where a series of redox reactions produces a proton gradient that drives ATP synthesis, using oxygen as the terminal electron acceptor (Ahmad et al. 2024). Meanwhile, at the molecular level, nicotinamide adenine dinucleotide or NAD can exist in either of two forms: the oxidized form-NAD^+^ and the reduced form-NADH. It is a dinucleotide combined through phosphate groups. One nucleoside contains an adenine base; the other contains nicotinamide. At the molecular level, NAD⁺ acts as an oxidizing agent in metabolic redox reactions, accepting electrons to become reduced and yield NADH according to Reaction 1: RH₂ + NAD⁺ → R + H⁺ + NADH. Where R is a reactant, for example sugar. NADH-produced by processes such as glycolysis and the citric acid cycle, therefore enters the ETC at Complex I. In the course of its entry into the ETC, NADH is responsible for translocating a total of 10 protons (H⁺): 4 protons in Complex I, 4 protons in Complex III, and 2 protons in Complex IV. The protons contribute to the electrochemical gradient across the inner mitochondrial membrane, which in turn drives ATP synthesis by means of ATP synthase. For every 4 H⁺ ions passing through ATP synthase, 1 ATP is generated, for a final yield of 2.5 ATP per NADH; this value is usually approximated as 3 ATP by many authors. When NADH is oxidized, it breaks down into NAD⁺, a proton H⁺, and two electrons e⁻ as in Reaction 2: NADH → H⁺ + NAD⁺ + 2e⁻. This transfer of electrons initiates the electron transport process, fueling the production of ATP in the mitochondria (Ahmad et al. 2024).

Mitochondrial biogenesis is the coordinated modulation of nuclear gene expression, protein import, and mtDNA transcription, which contributes to the extension of the organelle reticulum into broader membranous networks. This mitochondrial network, or reticulum, is evident amongst myofibrils and is beneficial for distributing mtDNA, proteins, and metabolites inside the myocyte’s depths (Carter et al. 2015; Jackson et al. 2022; Rahman and Quadrilatero 2021). Meticulous cellular communication coordination is required to ensure the proper stoichiometry of nuclear and mitochondrially encoded proteins that make up the organelle (Carter et al. 2015; Rahman and Quadrilatero 2021). Transcription results in the export of mRNAs from the nucleus, which are then translated into precursor proteins in the cytosol. Cytosolic chaperones guide them to the mitochondria, where they are imported through the protein import machinery (PIM). Once within, the proteins may be inserted into the organelle’s multiple compartments or function as transcriptional regulators for mtDNA, such as Tfam. When regions of the mitochondrial reticulum lose their membrane potential, and hence their capacity for ATP provision, or when ROS production is high, fission is activated, leading to cleavage of the dysfunctional segment of the organelle network (Table 1). Removal and degradation of this dysfunctional portion, mitophagy, is essential to maintain a healthy population of organelles within the muscle (Akbari et al. 2019; Fang et al. 2019; Onishi et al. 2021; Rahman and Quadrilatero 2021). This catabolic process requires recognizing the organelle segment to be digested and its subsequent enclosure in a double membrane autophagosome. Autophagy is the process of cytoplasmic material from both endogenous and exogenous origins, such as protein aggregates and organelles, being guided to lysosomes for degradation (Akbari et al. 2019; Galluzzi et al. 2017; Jackson et al. 2022; Rahman and Quadrilatero 2021). The autophagosome then merges with the lysosome, where proteolytic enzymes dissolve the contents. Pathology and the preservation of muscle mass have been attributed to deficiencies or mutations in the autophagy pathway, which result in an inability to efficiently eliminate defective mitochondria (Carter et al. 2015; Galluzzi et al. 2017; Gioran and Chondrogianni 2020; Rahman and Quadrilatero 2021).

### Mitochondrial homeostasis in muscle ageing

Mitochondria are the main sites for the processing of cellular ATP, as well as apoptosis (Ahn et al. 2022; Akbari et al. 2019; Pharaoh et al. 2021), cytoplasmic calcium buffering (Akbari et al. 2019; Reggiani and Marcucci 2022) and ROS-mediated signaling pathways (Akbari et al. 2019; Gerald & Tamas 2015; Jackson et al. 2022; Pharaoh et al. 2021). An age-related increase in oxidative stress-mediated by increases in ROS is one process that, if not matched by the activity of endogenous antioxidants, could cause mitochondrial dysfunction by damaging cellular lipids, proteins, and nucleic acids (Ahn et al. 2022; Jackson et al. 2022; Johnson et al. 2013). One of the main cellular ageing theories establishes a strong positive correlation between age and oxidative damage. Increased oxidative stress causes early-onset sarcopenia characterized by a breakdown in neuromuscular innervation, muscle atrophy, and mitochondrial muscle dysfunction (Pharaoh et al. 2021). The literature on human skeletal muscle strongly supports a positive correlation between age and oxidative damage to lipid peroxidation levels, protein carbonyl content, and 8-oxo-deoxyguanosine (8-oxo-dG) a measure of DNA oxidation. mtDNA is thought to be sensitive to oxidative damage due to its lack of histones and proximity to ROS produced by the ETC. Fazelzadeh et al. (2016) and Pharaoh et al. (2021) have found that ageing affects muscle glucose and fatty acid metabolism. Ageing also causes muscle group-specific disruptions in lipid and glucose metabolism, which are linked to mitochondrial dysfunction. These changes in lipid content and oxidative activity in skeletal muscle during ageing are associated with a shift in muscle fibre composition (Fazelzadeh et al. 2016; Pharaoh et al. 2021).

The location of mitochondrial genes, the diverse structure of mitochondria, bidirectional mitochondria-nucleus signaling, and pathways for the destruction of defective mitochondria contribute to a complex and integrated network that is critical for normal mitochondrial and cellular functions (Ahn et al. 2022; Akbari et al. 2019; Fakouri et al. 2019; Zhang and Vijg 2018). Evidence indicates that DNA damage and mutation accumulate in multiple human and animal tissues with age. Particularly, older individuals have lower expression levels of genes involved in mitochondrial activity than younger individuals. The age-related differences in gene expression levels in muscle are expected to be reflected in levels of metabolites. mtDNA is exclusively maternally inherited, and with time, mtDNA mutation will continue multiplying (Akbari et al. 2019; Area-Gomez et al. 2019; Grünewald et al. 2019). The proportion of mutant to normal mtDNA affects mitochondrial function and determines the severity and the progression of the disease and tissue dysfunction (Akbari et al. 2019; Chow and Herrup 2015; Kalfalah et al. 2015; Li et al. 2016, 2017; López-Otín et al. 2013; Mattson and Arumugam 2018; Maynard et al. 2015; Zhang et al. 2015). Because the mitochondrial inner membrane is a major site of mitochondrial ROS production, oxidative lesions are expected to occur frequently in mtDNA (Akbari et al. 2019; Marzetti et al. 2013). Indeed, mtDNA abnormalities increase with age and are associated with sarcopenia.

Another significant mechanism linking mitochondrial dysfunction to sarcopenia is the gradual decrease in ATP levels, which impacts protein synthesis and muscle growth as we age (Paulussen et al. 2021). ATP is the cell’s main source of energy, powering nearly all vital processes, including muscle repair and maintenance. As ageing progresses, the systems designed to maintain ATP levels struggle to keep up, leading to bioenergetic failure (Table 1). This breakdown creates a cycle of oxidative damage, depletion of mitochondrial DNA (mtDNA), and a decline in the ability of cells to maintain mitochondrial quality (Jackson et al. 2022; Li et al. 2017; Marzetti et al. 2013). A crucial regulator of this balance is AMPK, which serves as the cell’s energy sensor. AMPK plays a key role in managing energy by triggering processes like autophagy and mitophagy—systems that remove damaged mitochondria—and by slowing down energy-intensive processes such as protein and lipid production (Ahn et al. 2022; Herzig and Shaw 2018). When activated, AMPK phosphorylates ULK1 (Table 2), starting the turnover of damaged mitochondria, a process that has been observed in human cells and model organisms like *C. elegans* (Egan et al. 2011). Moreover, AMPK can promote mitochondrial fragmentation by activating mitochondrial fission factor (MFF), which helps the cell clear out faulty mitochondria (Gioran and Chondrogianni 2020; Toyama et al. 2016). However, as people age, AMPK activity naturally declines, which could be one of the reasons why mitochondrial function and biogenesis decrease (Burkewitz et al. 2014; Correia-Melo et al. 2016; Reznick et al. 2007). Evidence points to decreased in mitochondrial respiratory enzymes, especially complex IV, along with a reduction in mtDNA content and the activity of enzymes in the tricarboxylic acid cycle. This decline contributes to the impaired energy production seen in ageing muscle (Del Campo et al. 2018; Fang et al. 2017). Reduced AMPK activity during ageing weakens the cell’s ability to remove damaged mitochondria, worsening mitochondrial dysfunction.

**Table 2:** The role of mitochondria in sarcopenia (in vitro study)

Authors & References	Involvement of Mitochondria	Findings
Balsa et al. (2019)	ER stress (ERS) response	• Protein glycosylation perturbation may cause ERS as shown in vitro via glucose deprivation or tunicamycin-mediated block of protein glycosylation. The ERS response has been shown to be followed by enhancement of the mitochondrial complex activities, increased levels of super complex formation and cristae rearrangement in human osteosarcoma cells.
		• These findings reveal how a nutrient imbalance may be translated into a proteostatic threat that eventually requires the action of mitochondria to restore cellular homeostasis.
Bouman et al. (2011)	ER stress (ERS) response	• Another study showed that ERS induced mitochondrial fragmentation and this effect could be prevented by Parkin overexpression.
		• Recent evidence suggests Parkin is crucial between UPR and mitochondria as it plays a central role in mitochondrial dynamics, bioenergetics and mitophagy. Parkin upregulation during ERS was shown to be protective for mitochondria while its regulation by the PERK arm of UPRER was revealed.
		• In SH-SY5Y cells, mitochondrial depolarization following CCCP (Carbonyl cyanide m-chlorophenyl hydrazone) treatment, which uses a chemical compound that acts as a mitochondrial uncoupler that induced UPRER as suggested by the enhanced mRNA levels of Binding Immunoglobulin Protein (BiP), an ER chaperone.
Heo et al. (2010)	Interventions of ubiquitin-proteasome system (UPS)	• The mitochondria associated degradation or MAD pathway is a well-established connection between UPS and mitochondria.
		• By affecting the biogenesis of mitochondrial proteins, the cytosol-residing proteasomes have a massive impact on mitochondrial homeostasis and together with the mitochondrial proteases, form a complete protein quality control system.
		• During MAD, Cdc48, a UPS component, is recruited to stressed mitochondria via its interacting protein, Vms1, that is evolutionarily conserved in eukaryotes. The recruited Cdc48 together with its cofactor Npl14, extract ubiquitinated proteins from the outer mitochondrial membrane and present them to the proteasome for degradation.
Suhm et al. (2018)	Interventions of ubiquitin-proteasome system (UPS)	• A genetic screen in *Caenorhabditis elegans *showed that mutations in three genes encoding for proteins involved in various mitochondrial functions negatively affected proteasome activity without altering ATP production.
		• Inhibition of any of the mitochondrial respiratory complexes I, II, III and IV also resulted in proteolytic blockade in the cytosol.

In contrast, mTOR (mechanistic target of rapamycin) signaling primarily supports anabolic processes, especially through mTOR complex 1 (mTORC1), which responds to the availability of nutrients, growth factors, and energy (Akbari et al. 2019; Saxton and Sabatini 2017). mTORC1 promotes the synthesis of proteins and lipids necessary for growth and repair. However, in ageing muscles, prolonged mTORC1 activity inhibits autophagy, leading to mitochondrial dysfunction and muscle degradation (Akbari et al. 2019; Barja 2013). While AMPK fosters catabolic activities to restore energy, mTORC1 drives anabolic processes when energy is abundant. The balance between these two systems is critical as AMPK acts as a counterbalance to mTORC1 during low-energy conditions by activating TSC2, an inhibitor of mTORC1, and directly inhibiting raptor, a component of the mTORC1 complex (Inoki et al. 2003). This helps conserve energy and supports the removal of damaged mitochondria through autophagy. With ageing, as AMPK activity drops, unchecked mTORC1 activity inhibits autophagy, preventing the removal of damaged mitochondria and further exacerbating mitochondrial damage. Studies show that inhibiting mTORC1 can extend lifespan in model organisms by promoting autophagy, improving mitochondrial health, and reducing unnecessary protein synthesis (Akbari et al. 2019; Khan et al. 2017). Additionally, the suppression of mTORC1 has been shown to improve the selective removal of damaged mitochondria through mitophagy. As a result, the decline in AMPK and continued high levels of mTORC1 activity during ageing seem to contribute to the mitochondrial dysfunction that underlies sarcopenia (Burkewitz et al. 2014; Wiley et al. 2016). Furthermore, the interplay between the mitochondria and the endoplasmic reticulum (ER), known as mitochondria-associated membranes (MAMs); facilitates the transfer of lipids and calcium between these organelles. This communication system can be disrupted by nuclear DNA damage (Fang et al. 2019), mtDNA damage (Chae et al. 2018) and mitochondrial stress, such as the buildup of misfolded proteins (López-Otín et al. 2013; Zhao 2002). Overall, the shift toward decreased AMPK activity and prolonged mTORC1 signaling accelerates mitochondrial damage, leading to the metabolic impairments and muscle loss seen in ageing.

The key mediators of the mitochondrial retrograde signaling pathways include ROS, Ca^2+^, and the cellular AMP/ATP ratio. Elevated levels of ROS are generated in abnormal mitochondria. However, ROS are also produced under normal physiological conditions and can generate oxidative damage to cells and tissues and has long been considered a significant driving force in the ageing process, a concept known as the “free radical theory” of ageing (Ahn et al. 2022; Akbari et al. 2019; Jackson et al. 2022). Most biological macromolecules highly interact with ROS, which results in their oxidative alteration and eventually loss of function (Di Meo and Venditti 2020). Among the species that make up ROS are the hydroxyl radical, whose reactivity is so strong that it reacts relatively close to where it was formed, and other species, such as superoxide and hydrogen peroxide, which are less reactive. Thus, nitrogen-containing species, which are now indicated as reactive nitrogen species (RNS), include nitric oxide, relatively unreactive, and its derivative the peroxynitrite, a powerful oxidant, able to damage many biological molecules (Di Meo and Venditti 2020). Whole-body overexpression of catalase in the mitochondria of mice prevents the deleterious metabolic impairments associated with ageing. Interestingly as found in the studies by Calvani et al. (2013) and Joseph et al. (2016), older humans with sarcopenia have similar contents of endogenous antioxidants like superoxide dismutase, catalase, and glutathione peroxidase which parallels with the findings in ageing mice with whole-body overexpression of catalase in the mitochondria, compared to younger individuals, suggesting that more endogenous antioxidant activity is necessary to combat the age-associated increase in ROS production. In both cases, the presence of these antioxidants appears to play a role in mitigating the metabolic impairments associated with ageing.

Skeletal muscle mitochondrial OXPHOS capacity can be measured in vivo and non-invasively in humans via phosphorus-31 magnetic resonance spectroscopy (_31_PMRS) (Choi et al. 2016; Gonzalez-Freire et al. 2018; Kumar et al. 2017; Marzetti et al. 2013). Both resting and maximal oxygen consumption decreases with advancing age. This decline is independent of fat-free mass, indicating that either muscle mitochondrial function or content are reduced as a function of age (Gonzalez-Freire et al. 2018; Marzetti et al. 2013). Studies on muscle specimens from healthy individuals have revealed age-related declines in mitochondrial mass, activities of tricarboxylic acid cycle enzymes, oxygen consumption (Coen et al. 2013; Gonzalez-Freire et al. 2018) and ATP synthesis (Choi et al. 2016; Gonzalez-Freire et al. 2018). The impact of mitochondrial bioenergetic decline on muscle ageing correlates with ATP synthesis, oxygen consumption, and preferred walking speed in healthy elderly (Choi et al. 2016; Coen et al. 2013; Gonzalez-Freire et al. 2018). Moreover, walking speed has been used in research as one of the ways to define bioenergetics and sarcopenia. A decrease in oxidative phosphorylation (OXPHOS) activity has been detected in vivo via _31_P nuclear magnetic resonance (_31_P-NMR) spectroscopy in older persons compared with younger controls (Choi et al. 2016; Gonzalez-Freire et al. 2018; Kumar et al. 2017; Marzetti et al. 2013). A study by Ahn et al. (2022) uses peroxiredoxin 3 (Prdx3), the primary antioxidant enzyme that scavenges hydrogen peroxide and is shown to be exclusively expressed in the mitochondrial matrix against a mouse model of redox-dependent sarcopenia, mice lacking superoxide dismutase 1 (Sod1). The study found that increased mitochondrial hydrogen peroxides in Sod1KO were reduced by mPRDX3 upregulation. Complex I‐ and II‐activated OXPHOS capacities in the Sod1KO mouse model were also significantly increased by overexpression of mPRDX3. Metabolic syndrome has been shown to disrupt mitochondrial function, depress skeletal muscle OXPHOS, and lead to ectopic lipid storage in skeletal muscle (Kumar et al. 2017). Whether acquired or genetic, aberrant mitochondrial function could contribute to the development or progression of cardiac dysfunction. Decreased OXPHOS can also arise from genetic defects. Friedrich’s ataxia (FA), the most common form of hereditary ataxia, is a genetic disorder caused by a mutation in the frataxin (FXN) gene, resulting in intra-mitochondrial iron accumulation, reactive oxygen species production, and abnormalities of oxidative phosphorylation (Kumar et al. 2017). Under pressure overload and heart failure conditions, the primary energy substrate preference switches from free fatty acids (FFA) to glucose. This is associated with decreased ETC activity and oxidative phosphorylation. Diabetes and metabolic syndrome result in mitochondrial abnormalities in the myocardium, such as impaired biogenesis and fatty acid metabolism, which lead to reduced substrate flexibility, energy efficiency, and, eventually, diastolic dysfunction (Kumar et al.^ 2017^).

Several recent studies have identified a link between nuclear DNA damage, cell metabolism, and mitochondrial homeostasis through NAD^+^ consumption. NAD^+^ is an important redox coenzyme in metabolic pathways such as the citric acid cycle and glycolysis. It is also used as a co-substrate by three classes of enzymes: (1) - poly (ADP-ribose) polymerases (PARP1/2), (2) - sirtuins, and (3) - the cyclic ADP-ribose (cADPR) synthases (CD38, CD157) (Cantó et al. 2015). Increased PARP activity, because of prolonged genotoxic exposure, defects in DNA repair, and during the ageing process, limit the level of NAD^+^ available for sirtuins, consequently affecting the function of their downstream targets such as peroxisome proliferator-activated receptor G coactivator-1α (PGC1-α), an important regulator of mitochondrial biogenesis and proteins involved in mitophagy. Accordingly, NAD^+^ supplementation and PARP inhibition increase cellular NAD^+^ levels and improve mitochondria homeostasis in various animal models of human diseases (Akbari et al. 2019; Cantó et al. 2015; Fang et al. 2019; Fang et al. 2017).

Del Campo et al. (2018) focus on the close link between Ca^2+^ and excitation-contraction (EC)-coupling during the ageing process, particularly in old rodent models. Impaired EC coupling function has been described in aged muscle resulting in a reduced supply of Ca^2+^ ions to the contractile elements and, thus, reduced specific force. These findings correlate with previous studies on impaired Ca^2+^ release in aged muscle and reduced sarcoplasmic reticulum (SR) Ca^2+^ release in SR vesicles. In addition, other defects, such as uncoupling between the voltage sensors, may also contribute to muscle weakness in ageing. Age-dependent uncoupling of mitochondria from the Ca^2+^ release units was proposed. They described an increase in damaged mitochondria together with reorganization towards the SR, with a significant decrease in tethers, which were displaced from their normal triad position, possibly resulting in reduced metabolic efficiency and a consequent decline in skeletal muscle performance. Del Campo et al. (2018) also demonstrated alterations in mitochondrial Ca^2+^ handling as a function of age. We see an increase in mitochondrial fusion and size, repositioning mitochondria to maximize contact with SR and complete buffering of IP3-dependent cytosolic Ca^2+^ by mitochondria in adults. Together with this adaptive process, mitochondria resting Ca^2+^ levels dramatically drop in the adult group compared to the young and the juvenile muscle, whether these changes in resting Ca2^+^ are related to the morphological changes seen at this age.

### Mitochondria Instability in Sarcopenia

**Fig. 1:**
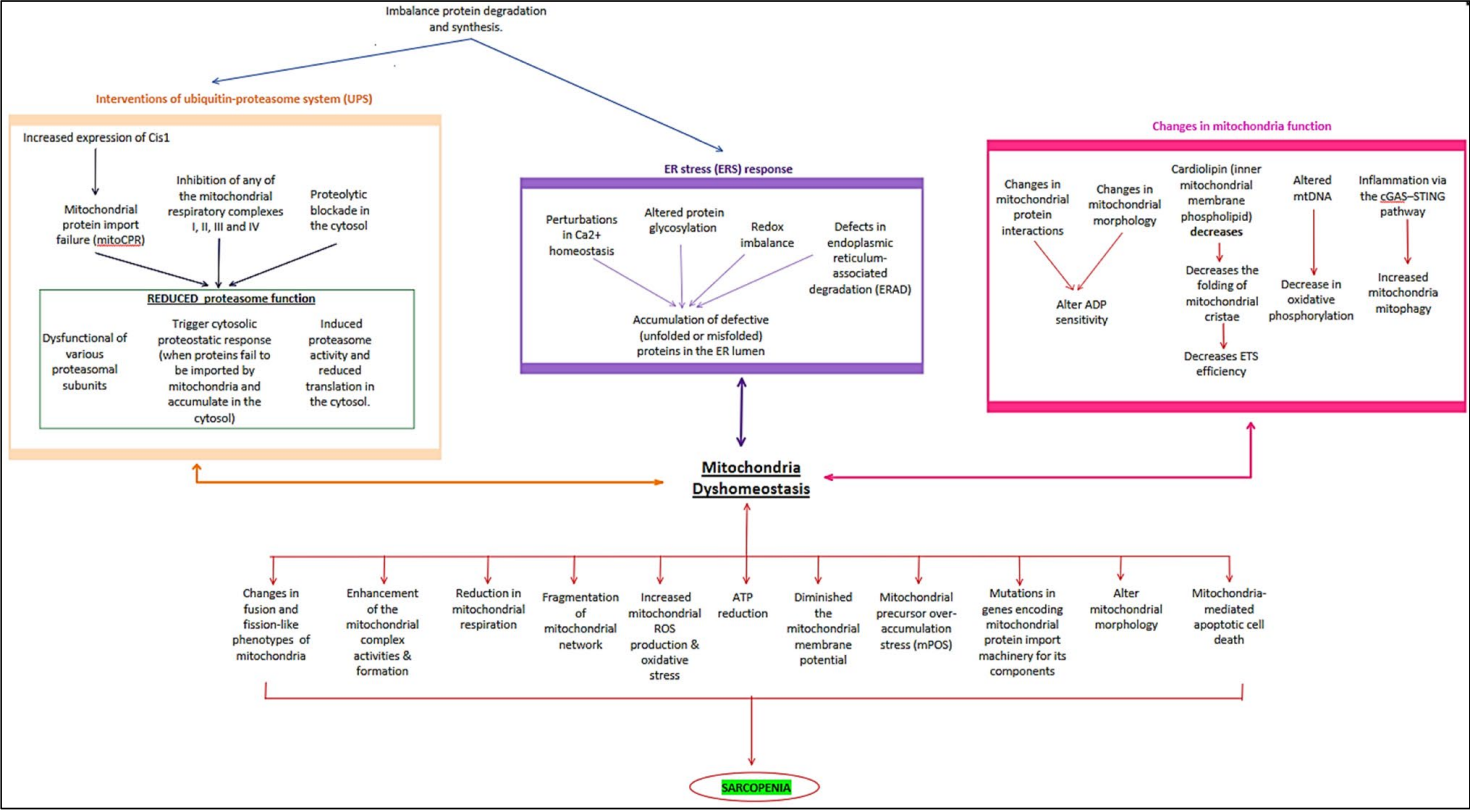
The role of mitochondria dyshomeostasis in sarcopenia

Mitochondria have been suggested as possible sarcopenia mediators, and the dysfunction of mitochondria contributes majorly to the symptoms of ageing (Ahn et al. 2022; Akbari et al. 2019; Carter et al. 2015; Del Campo et al. 2018; Jackson et al. 2022; Pharaoh et al. 2021). Figure 1 shows the various pathways of involvement of mitochondria that lead to sarcopenia. Mitochondria are needed for basal cellular and organismal function, and any defects in the pathways that regulate mitochondrial DNA (mtDNA) maintenance and function are pathogenic, which will directly affect muscle functionality (Akbari et al. 2019; Del Campo et al. 2018; Jackson et al. 2022; Pharaoh et al. 2021). Subtle changes and defects in these pathways will affect mitochondrial homeostasis and function and have been attributed to age-related cognitive deficits, sarcopenia (Marzetti et al. 2013), insulin tolerance (Akbari et al. 2019; Timothy et al. 2011), and brain ageing (Akbari et al. 2019; Mattson and Arumugam 2018). Hence, the mitochondrial activity of the skeletal muscle is critical to health and deterioration in different mitochondrial factors can be observed in muscle as people get older. Most of this deterioration is due to age-related changes in mitochondrial synthesis and degradation (Carter et al. 2015; Del Campo et al. 2018; Pharaoh et al. 2021). Previous research has proved that giant and unstable mitochondria are heavily intertwined in ageing skeletal muscle, and anomalies can be seen in their ultrastructure. These findings have contributed to the hypothesis that ageing causes an imbalance in mitochondrial dynamics in musculoskeletal tissue (Carter et al. 2015; Del Campo et al. 2018; Pharaoh et al. 2021).

Moreover, related fibre forms have shown different plays in mitochondrial networks and glycolytic enzymes (Del Campo et al. 2018). Both the distribution and orientation of mitochondria in muscle fibres changed with age. The Del Campo et al. (2018) study on mice observed the most notable change occurring at 10 to14 months old, where a redistribution of mitochondria occurs towards a more striated pattern, oriented perpendicularly to the fibre's longitudinal axis. In mice older than 14 months, an increased presence of longitudinal orientation of mitochondria is observed, though the striations are less defined compared to adult fibres, with some mitochondria forming longitudinal columns between the myofibrils (Del Campo et al. 2018). Pathak and Trebak (2018) also demonstrated that non-linear changes in mitochondrial fusion and fission-like phenotypes and the orientation of mitochondria correlate with changes in the SR-mitochondria communication and mitochondrial Ca^2+^ handling. These changes also appear to correlate with changes in muscle function evidenced at the different stages of mice’s lives. These findings strongly suggest that mitochondrial topology and morphology are involved in the process of sarcopenia and loss of muscle function during ageing, which appears to start before old age.

Mitochondria also play various pivotal roles in regulating innate and adaptive immune responses (Samuel et al. 2015; Weidberg and Amon 2018). Thus, the beneficial health effects of lifestyle and compounds that stimulate mitophagy and maintain mitochondrial quality (Fang et al. 2017, 2019) are likely to control chronic inflammation, which is a major source of age-related sarcopenia, in which directly impact to frailty and morbidity (Akbari et al. 2019; Franceschi et al. 2017; López-Otín et al. 2013; Mattson and Arumugam 2018). The inflammatory response is triggered by the release of mediators such as cytokines and chemokines by tissue-resident leukocytes and other cells (Akbari et al. 2019; Sliter et al. 2018). The cyclic guanosine monophosphate-adenosine monophosphate synthase (cGAS) and stimulator of interferon genes (STING) signaling has been identified as a key sensor of cytoplasmic DNA. The release of mtDNA into the cytoplasm, as a result of mitochondrial stress and damage, can activate the cGAS-STING inflammatory response (Akbari et al. 2019; Michael et al. 2014; Sliter et al. 2018; West et al. 2015) but the removal of damaged or stressed mitochondria by mitophagy inhibited this response (Akbari et al. 2019; Sliter et al. 2018).

Apoptosis expresses myonuclear deterioration, which is a potential mechanism contributing to sarcopenia. Mitochondria regulates apoptosis as ROS emission from the ETC can induce the mtPTP to open, affecting the amount of membrane potential, a reduction in ATP production, and the swelling of organelles (Ahn et al. 2022; Pharaoh et al. 2021). Upon the release of pro-apoptotic proteins such as cytochrome c, apoptosis-inducing factor (AIF), and endonuclease G (Endo G) into the cytoplasm, DNA fragmentation occurs either in a caspase-dependent (cytochrome c) or caspase-independent mechanism (AIF, Endo G). Compared to young muscle, ageing muscle produces more mitochondrial ROS. It has less calcium retained, which causes the organelle to release more cytochrome c and Endo G. As a result, the rate of myonuclear DNA fragmentation is almost three times higher in ageing muscle. If restricted to a certain area of the fibre, nuclear decay could lead to regional atrophy and the possible disappearance of this fibre segment. Chronic contractile activity can completely reverse this myonuclear decay in ageing muscle. Indeed, regular exercise can increase anti-apoptotic Bcl-2 levels, reduce apoptotic protein release from mitochondria, and decrease DNA fragmentation, illustrating at least one mechanism through which exercise can potentially preserve muscle mass through improved mitochondrial function (Carter et al. 2015).

The close relationship between ageing, mitochondrial function, and metabolic disorders has received much attention. Three proteins have emerged as key metabolic signals: (1) PGC-1α, (2) silent mating-type information regulation 2 homolog sirtuin 1 (SIRT1), and (3) AMP-activated protein kinase (AMPK) (Johnson et al. 2013). PGC-1α can stimulate the formation of new mitochondria in cell culture and is an important regulator of exercise tolerance and mitochondrial adaptations to aerobic exercise in rodents (Gu et al. 2021; Johnson et al. 2013; Yang et al. 2020) and humans (Gu et al. 2021; Safdar et al. 2010). Overexpression of PGC-1α in the skeletal muscle of mice attenuates mitochondrial dysfunction at 22 months of age (Wenz et al. 2009; Yang et al. 2020). Thus, PGC-1α has an important role in regulating mitochondrial proteostasis by promoting the expression of new mitochondrial proteins and maintaining protein health in response to stress, such as ageing or exercise. A study by Dillon et al. (2012) also showed that increased expression of PGC-1α in the skeletal muscle of ageing wild-type (WT) mice protects from mitochondrial dysfunction and sarcopenia associated with normal ageing (Dillon et al. 2012). A key regulator of mitochondrial biogenesis is PGC-1α. Overexpression of PGC-1α in the mouse skeletal muscle and heart has been shown to increase mitochondrial biogenesis and function (Dillon et al. 2012). In addition, PGC-1α induces a fibre-type switch from glycolytic to oxidative fibres, angiogenesis and retards protein degradation and atrophy in the skeletal muscle. PGC-1α has been shown to regulate the conversion of muscle fibres from glycolytic (type IIB) to oxidative (type I and IIA) fibre types. Some groups have reported lower skeletal muscle PGC-1α content between age groups in humans aged around 70 years (mRNA) and rats aged 36 months (protein), but not in mice aged 24 months (mRNA and protein). Interestingly, endurance exercise training promotes PGC-1α protein content in skeletal muscle of 34-month-old rats and humans aged 59–76 years. Hence, understanding how the ability of PGC-1α to regulate the health of mitochondrial proteins changes with age is crucial (Johnson et al. 2013; Yang et al. 2020).

One mechanism by which AMPK increases energy production is through increased mitochondrial biogenesis. Decreased basal activation of AMPK may contribute to the loss of mitochondrial content with age. However, with ageing, the basal activation of AMPK declines, which may contribute to the loss of mitochondrial content. Additionally, the response of AMPK to nutrient or exercise stimuli weakens over time, as evidenced by older rats showing reduced AMPK activity following nutrient intake or aerobic exercise (Reznick et al. 2007). This diminished signaling can lead to lower mitochondrial protein synthesis, resulting in the accumulation of damaged mitochondrial proteins. While AMPK promotes mitochondrial growth during energy stress, mTOR serves a different function. It is a well-known pathway that governs cell growth and metabolism based on nutrient availability and energy levels. Operating through two complexes, mTORC1 and mTORC2, it encourages anabolic processes such as protein synthesis when resources are sufficient (Akbari et al. 2019; Saxton and Sabatini 2017). However, as we age, the sustained activity of mTORC1 in muscle may hinder autophagy and continue promoting protein synthesis, even when energy levels are low. This sets up a delicate balance between AMPK and mTORC1. Under normal conditions, AMPK acts as a safeguard, conserving energy during low-energy states by inhibiting mTORC1, while mTORC1 drives growth when resources are plentiful. However, as we age, the decline in AMPK activity reduces our ability to maintain mitochondrial health, further worsening mitochondrial dysfunction and contributing to age-related conditions like sarcopenia. Ultimately, the interplay between mTORC1’s continued push for growth and AMPK’s diminishing response to energy stress underscores how our cellular energy regulation becomes less effective over time. This shift in balance not only complicates our metabolism but also highlights the metabolic challenges associated with ageing, illustrating the need for a deeper understanding of these pathways to develop strategies for promoting healthier ageing.

### Mitochondrial transcriptomic and proteomic in Sarcopenia

The study that mtDNA mutations alter several mitochondrial quality control processes, including biogenesis, fusion/fission, and autophagy, and that this is likely one of the underlying mechanisms contributing to sarcopenia in the model of premature ageing. Importantly, the findings are also relevant to normal ageing since mitochondrial mutation rates steadily increase in normally aged muscle (Ahn et al. 2022; Gioran and Chondrogianni 2020; Joseph et al. 2016). In sedentary individuals, mitochondrial content declines with age when measured as enzyme activities or quantitative proteomics (Johnson et al. 2013). Protein aggregates will develop as a result of proteostasis or protein homeostasis. A complex network of molecular chaperones, proteolytic machinery, and their regulators, known as the proteostasis network, occurs in healthy cells (Hipp et al. 2019). These elements integrate the synthesis of proteins with polypeptide folding, protein structure conservation, and protein degradation. Nevertheless, maintaining proteome balance is difficult due to several environmental and internal pressures that pile up with ageing. The capacity and integrity of the proteostasis network are reduced due to these stresses. The resulting accumulation of misfolded and aggregated proteins affects postmitotic cell types such as neurons, manifesting in disease (Hipp et al. 2019). Genes involved in DNA repair, cell death, transcription, and cell cycle were upregulated, while genes associated with mitochondrial function and metabolism were downregulated with age. Phillips et al. (2013) have collected a list of 500 genes that track with age and differentiated these genes from those that react to different types of physical activity. Although the findings of these studies are significant for discovering new pathways associated with ageing, it should be noted that changes in mRNA are not often expressed in protein levels. There has been a greater divergence in findings where proteomic methods have been used. Théron et al. (2014) discovered 35 proteins that changed in expression with age, most of which were downregulated and active in energy synthesis, myofilament, or the cytoskeleton.

Most proteomic studies found declines in essential mitochondrial enzymes in ageing muscle, according to Lourenço Dos Santos et al. (2015). However, inconsistency in protein expression may be observed due to the form of sample extract used in the study, which could be whole muscle or isolated mitochondria (Akbari et al. 2019; Carter et al. 2015; Fang et al. 2019; Jackson et al. 2022; Onishi et al. 2021; Pharaoh et al. 2021). The first study to measure mitochondrial protein synthesis rates in young, middle-aged, and older individuals in vivo was by Rooyackers et al. (1996). A previous study on animal model shows that 40% of protein synthesis rate decreased until the rodents reached middle age (Johnson et al. 2013). Subsequent measurements in humans of mitochondrial protein synthesis in response to amino acids and insulin found a reduced stimulatory response in the elderly, possibly due to a defect in phosphorylation of S6K1 (Johnson et al. 2013). Surprisingly, with 6 months of strength exercise, 179 of the 596 genes correlated with age were normalized toward a younger transcriptomic signature (Carter et al. 2015; Groennebaek and Vissing 2017).

Protein degradation by the autophagy-lysosome and ubiquitin-proteasome pathways is important to maintain mitochondrial function in muscles and fits a model that decreased protein turnover with age leads to the accumulation of damaged proteins with decreased function. Mitochondrial proteins are particularly susceptible to damage due to their proximity to ROS formation. The ubiquitin-proteasome and Lon protease pathways (Table 2) catalyze the selective removal of damaged proteins within the mitochondria. In contrast, the autophagy-lysosome pathway degrades larger volumes of mitochondrial proteins or membranes via mitophagy. A decrease in proteolysis pathways with age has been detected in various tissues, including Drosophila flight muscle (Tsakiri et al. 2019), mouse liver (Ugun-Klusek et al. 2017), rat skeletal muscle (Jeong et al. 2024; Safdar et al. 2010; Song et al. 2021) and human fibroblasts. Transgenic model systems revealed that increasing autophagy-related proteins could improve mitochondrial function during mitochondrial damage or ageing. Damaged mitochondrial proteins are removed by mitophagy in human umbilical vein endothelial cell cultures (Onishi et al. 2021), and overexpression of ATG5, ATG7, and LC3B protected mitochondrial function following ROS-induced damage (Johnson et al. 2013; Rahman and Quadrilatero 2021; Rodger et al. 2018; Tsakiri et al. 2019). Further, transgenic mice that maintain the autophagy protein lysosome-associated membrane protein 2A (LAMP2) in the liver have restored autophagy and mitochondrial function with age (Table 2) compared to wild-type mice (Liu et al. 2020; Zhang and Vijg 2018). AMPK activation restricts global protein synthesis and skeletal muscle cell growth by inhibiting the mammalian target of rapamycin (mTOR) complex 1 (mTORC-1). However, AMPK activation also increases the mitochondrial enzyme content of skeletal muscle. Protein synthesis requires ATP and is decreased with AMPK activation. Thus, AMPK must inhibit global protein synthesis while simultaneously increasing mitochondrial protein synthesis. It is likely that AMPK coordinates with PGC-1α and sirtuin activity to increase mitochondrial protein synthesis selectively while decreasing overall protein synthesis during energy stress (Johnson et al. 2013). AMPK and SIRT1, two key metabolic sensors, have been shown to directly regulate PGC-1α activity through phosphorylation and deacetylation, respectively (Cantó and Auwerx 2009). Sirtuins, a family of proteins known for their role in delaying ageing and mitigating age-related diseases, exert their effects through multiple molecular pathways. These include promoting DNA damage repair, delaying telomere shortening, and mediating the longevity effects of caloric restriction (Wan et al. 2022). The sirtuin family (SIRT1-7) is evolutionarily conserved and functions as NAD^+^-dependent deacetylases, regulating various cellular metabolic processes by deacetylating specific target proteins (Wan et al. 2022).

A cross-sectional analysis of sedentary older individuals compared to active older and young individuals (Safdar et al. 2010) reinforces the results of others that mitochondrial function can be maintained as humans age. Interestingly, the manganese superoxide dismutase (MnSOD) enzyme isolated from sedentary older subjects was found to be highly damaged, as measured by nitration of tyrosine residues which may reduce the function of the enzyme. This suggests that the intracellular environment accumulates damage to proteins directly related to ameliorating oxidative stress (Johnson et al. 2013). Cellular senescence has been widely recognized as a distinctive and essential characteristic of ageing tissues (Akbari et al. 2019). It is characterized by a state of irreversible and permanent proliferation arrest in somatic cells, together with a complex and distinct senescence-associated secretory phenotype (SASP) consisting of upregulation of pro-inflammatory cytokines, proteases, and growth and angiogenesis factors that can alter tissue homeostasis. Senescence can be induced by various stress factors, including mitogenic stress, and persistent DNA damage, including telomeric DNA, and activation of DNA damage response (DDR), and is commonly thought to have been developed to counter oncogenic transformation of the affected cell (Wiley et al. 2016; Zhang and Vijg 2018). Over time, the number of senescent cells increases, resulting in diminished tissue function, altered tissue structure, and a decline in tissue repair and regenerative capacity, collectively promoting ageing and age-related phenotypes (Chapman et al. 2019). Cells treated with various DDR activators and senescence inducers display increased mitochondrial mass prior to cell cycle arrest (Chapman et al. 2019; Moiseeva et al. 2017). This is via transcriptional activation of PGC-1β, resulting in increased mitochondrial ROS production and DNA damage. Persistent DNA damage gives rise to a positive feedback loop through ATM, Akt, and mTORC1 phosphorylation activation, followed by enhanced PGC-1β-dependent mitochondrial biogenesis, sustained DDR, cell cycle arrest and cellular senescence (Correia-Melo et al. 2016; Summer et al. 2019). The ATM-Akt-mTORC1 pathway plays a key role in cellular senescence, particularly in response to persistent DNA damage. Ataxia-Telangiectasia Mutated (ATM), a protein kinase activated by DNA double-strand breaks, initiates the DDR, leading to cell cycle arrest and repair. When activated long-term, ATM promotes mitochondrial biogenesis via PGC-1β, increasing ROS production and reinforcing senescence features such as inflammation and metabolic dysfunction (Correia‐Melo et al. 2016; Stagni et al. 2021). Akt, a serine/threonine kinase, in tandem with ATM and mTORC1, helps regulate cellular metabolism and survival. Persistent activation of Akt-mTORC1 exacerbates senescence by further promoting mitochondrial activity and oxidative stress. (Stagni et al. 2021; Summer et al. 2019). Together, these pathways create a feedback loop that sustains the senescence state, contributing to age-related diseases. Interventions targeting mTORC1 or mitochondrial ROS could help mitigate the harmful effects of senescence. Mitochondrial dysfunction-associated senescence (MiDAS) is characterized by distinct SASP and reduced NAD^+^/NADH ratio and AMPK activation. Activated AMPK was shown to drive growth arrest and senescence through phosphorylation of p53 that increased the expression of its target senescence markers p21WAF1 and p16INK4a (Wiley et al. 2016). Senescence and MiDAS phenotypes were also detected in tissues from transgenic mice that accumulate mtDNA mutations and dysfunctional mitochondria and show accelerated ageing phenotypes, demonstrating MiDAS in vivo (Wiley et al. 2016). Thus, cellular senescence may be an important consequence of an age-related loss in mitochondrial homeostasis. Because of the key roles of senescence and mitochondrial dysfunction in ageing and age-related tissue dysfunction and disease, more in vivo studies in animal models and human tissues are needed. Collectively, current data suggest that cellular senescence integrates several regulators of longevity and health span, especially in genome instability and DDR, mitochondrial metabolism, AMPK activation, and mTOR pathway function, which are largely druggable targets, including senescent cells and SASP (Arriola Apelo et al. 2016; Bai et al. 2011; Kraig et al. 2018).

The mitochondria-associated degradation or MAD pathway is a well-established connection between UPS and mitochondria. During MAD, Cdc48, a UPS component, is recruited to stressed mitochondria via its interacting protein, Vms1, which is evolutionarily conserved in eukaryotes. The recruited Cdc48, together with its cofactor Npl14, extract ubiquitinated proteins from the outer mitochondrial membrane and present them to the proteasome for degradation (Heo et al. 2010). It was also found that cortical neurons from mice lacking a proteasome subunit manifested a fragmented mitochondrial network and respiratory complex I defects (Ugun-Klusek et al. 2017). The proteasome inhibitor lactacystin increased mitochondrial ROS production and oxidative stress and diminished the mitochondrial membrane potential in mouse cortical neurons (Ugun-Klusek et al. 2017). Wrobel et al. (2015) challenged the mitochondrial protein import machinery by mutations in genes encoding for its components, specifically the inner membrane translocase, TIM23. All insults induced proteasome activity and reduced translation in the cytosol. This response was named “unfolded protein response activated by mistargeted proteins” or UPRam, which was suggested to maintain cellular homeostasis. Notably, UPRam induction also rendered the cells more resistant to heat shock, thus possibly indicating the activation of an additional proteostatic response (Wrobel et al. 2015). Failed cytosolic proteostatic response occurs when proteins fail to be imported by mitochondria and accumulate in the cytosol. Wang and Chen (2015) used a yeast strain that carried a lethal mutation of a gene encoding for a mitochondrial adenine nucleotide translocase. The mutation caused aberrant accumulation of mitochondrial precursors in the cytosol; a phenomenon named mitochondrial precursor over-accumulation stress (mPOS). A different model of the cytosolic response was named mitochondrial compromised protein import response or mitoCPR (Weidberg and Amon 2018). It is described as a surveillance mechanism activated upon mitochondrial protein import failure. To cause mitochondrial import stress, the authors overexpressed the mitochondrial proteins. MitoCPR includes increased expression of Cis1, a translocase-interacting mitochondrial outer membrane protein, and the recruitment of the ATPases associated with diverse cellular activites (AAA^+^ ATPase) Msp1 by Cis1 that clears the import channels by marking the accumulated precursors for proteasomal degradation. Suhm et al. (2018) conducted a genetic screen in Caenorhabditis elegans, which showed that mutations in three genes encoding for proteins involved in various mitochondrial functions negatively affected proteasome activity without altering ATP production. Inhibition of mitochondrial respiratory complexes I, II, III and IV also resulted in a proteolytic blockade in the cytosol (Suhm et al. 2018). Accordingly, mice carrying a deletion of Ndufs4, a mitochondrial complex I subunit, exhibited reduced proteasome activities in their midbrain (Song and Cortopassi 2015). Table 1 provides the summaries of past studies regarding the involvement of mitochondria in sarcopenia in in-vivo study of human and animal, while Table 2 provides the summaries of past studies on involvement of mitochondria in sarcopenia in in-vitro study.

## Conclusion and future perspective

In conclusion, this review paper has provided an overview of the various factors and mechanisms involved in the pathogenesis of sarcopenia and muscle degeneration with age. We have highlighted the complex nature of sarcopenia, which is influenced by multiple factors ranging from age-related brain changes, muscle fibre denervation, imbalances in protein synthesis and degradation, and declining mitochondrial function. The paper emphasizes the detrimental effects of age-related changes on muscle physiology, leading to a decrease in muscle mass, strength, and functionality. These changes not only pose significant health risks for individuals but also impact their overall quality of life, independence, and mobility. Moreover, the paper has shed light on the role of oxidative stress, cytokines, and molecular processes such as protein turnover and mitochondrial dysfunction in the development and progression of sarcopenia. Understanding these underlying mechanisms is crucial for designing effective strategies to mitigate or reverse muscle impairment associated with ageing. However, it is important to acknowledge that there are still gaps in our understanding of sarcopenia and the interactions between different cellular processes involved. Further research is needed to explore and elucidate these mechanisms and potential therapeutic interventions to target age-related muscle degeneration. Overall, this paper contributes to the existing body of knowledge on sarcopenia and serves as a foundation for future research endeavors aimed at improving the prevention, diagnosis, and treatment of age-related muscle decline, ultimately enhancing the health and well-being of the ageing population.

## Data Availability

No datasets were generated or analysed during the current study.
